# Microporosity Clustering Assessment in Calcium Phosphate Bioceramic Particles

**DOI:** 10.3389/fbioe.2019.00281

**Published:** 2019-10-18

**Authors:** Raúl Vallejos Baier, Isabel Benjumeda Wijnhoven, Víctor Irribarra del Valle, Carola Millán Giovanetti, Juan F. Vivanco

**Affiliations:** ^1^Faculty of Engineering and Sciences, Adolfo Ibáñez University, Viña del Mar, Chile; ^2^Faculty of Liberal Arts, Adolfo Ibáñez University, Santiago, Chile

**Keywords:** calcium phosphate, bioceramic particle, microporosity, data mining, K-means clustering

## Abstract

There has been an increase in the application of different biomaterials to repair hard tissues. Within these biomaterials, calcium phosphate (CaP) bioceramics are suitable candidates, since they can be biocompatible, biodegradable, osteoinductive, and osteoconductive. Moreover, during sintering, bioceramic materials are prone to form micropores and undergo changes in their surface topographical features, which influence cellular physiology and bone ingrowth. In this study, five geometrical properties from the surface of CaP bioceramic particles and their micropores were analyzed by data mining techniques, driven by the research question: what are the geometrical properties of individual micropores in a CaP bioceramic, and how do they relate to each other? The analysis not only shows that it is feasible to determine the existence of micropore clusters, but also to quantify their geometrical properties. As a result, these CaP bioceramic particles present three groups of micropore clusters distinctive by their geometrical properties. Consequently, this new methodological clustering assessment can be applied to advance the knowledge about CaP bioceramics and their role in bone tissue engineering.

## Introduction

It is estimated that ~1.5 million devices are implanted worldwide per year to heal musculoskeletal diseases (Holzapfel et al., 2013), these procedures present difficulties that have economic implications for health care providers. Likewise, given the complications in the access or usage of grafts, there has been an increase in the application of different biomaterials in order to repair hard tissues, such as bone and teeth (Giannoudis et al., 2005).

One type of biomaterials currently used for hard tissue regeneration are calcium phosphate (CaP) based bioceramics, which not only have a similar composition to the mineral phase of bone (Dorozhkin, 2010), but can be biocompatible, biodegradable, osteoinductive, and osteoconductive (Vivanco et al., 2012; Denry and Kuhn, 2016; Kim et al., 2017). In addition, CaP bioceramics promote rapid bone formation and may assure bone healing within a year (Habraken et al., 2016). Moreover, it has been shown that calcium and phosphate ions trigger an osteoinductive response during bone regeneration, being resorbed by cell-mediated processes, therefore controlling the potential toxicity of degradation products (Habraken et al., 2016).

Recent advances have started to focus on using CaP bioceramics for bone tissue regeneration, given that they can be inserted in the defect site with a minimally invasive surgery, preventing the risks of infections, surgical scars, and blood loss (Low et al., 2010; Uswatta et al., 2016). Among CaP bioceramics, hydroxyapatite (HA) and tricalcium phosphate (TCP) have been commonly used in clinical applications and in *in vivo* studies. For example, some studies have shown that 95% of these CaP bioceramics are resorbed in 26–28 weeks (Knaack et al., 1998; Wiltfang et al., 2002), with crystalline TCP having a higher degradation rate than crystalline HA (Vicente et al., 1996; El-Ghannam, 2004). This degradation profile is a desirable property, given that it allows the replacement of the bioceramic material with newly synthetized bone.

Previous evidence suggests that the structural and material properties of a CaP bioceramic strongly influence its capacity to induce new bone formation (Habibovic and de Groot, 2007). Additionally, it is not only the material *per se* or its fabrication parameters which influence biological processes (Habraken et al., 2016), but also its final microarchitecture, such as pore size and pore distribution (Gauthier et al., 1998; Mastrogiacomo et al., 2006; Novotna et al., 2019). Correspondingly, the porosity of a bioceramic is an important requirement for vascularization (Karageorgiou and Kaplan, 2005).

Although there is a general agreement that pore size is a key factor affecting cell ingrowth and bone formation in bioceramics (Hutmacher, 2000), there is no conclusive data regarding optimal pore size. For example, a minimal pore diameter of 100 μm has been proposed to influence cell ingrowth (Karageorgiou and Kaplan, 2005), while a diameter of 200 μm or more has been proposed to support new bone formation (Gauthier et al., 1998; Flautre et al., 2001; Galois and Mainard, 2004). Interestingly, some *in vivo* studies have suggested that there is no significant difference in bone regeneration for pore sizes in the range of 400–1,200 μm (Hollister et al., 2005; Schek et al., 2006). However, there is evidence for bone formation in structures with interconnected micro-pores of <10 μm in size, while also having a macro-porosity of more than 100 μm (Lan Levengood et al., 2010a,b).

It is well-known that microporosity affects the process of osteogenesis (Zhang et al., 2018; Rustom et al., 2019), for instance, the increased specific surface areas by microporosity in biomaterials can offer more protein adsorption sites. Additionally, the capillary force generated by this microporosity can improve the attachment and immigration of bone-related cells on the biomaterial surface, with these cells penetrating them even if these micropores are smaller (Polak et al., 2013; Zhang et al., 2018). The contribution of capillarity induced by microporosity on biphasic CaP biomaterials has also been tested *in vivo*, by experiments with pig mandibles, which showed that capillarity induced by microporosity improved the homogeneity of bone distribution in these biomaterials (Rustom et al., 2016).

Although the above-mentioned features of a biomaterial, such as the specific surface area and the capillary force generated by its microporosity, have been shown to play a role in osteogenesis, detailed analyses of each of these features might not be yet fully quantified. Likewise, while properties such as pore size, localization, or gradients have been studied in relation to cell growth and bone formation, more global properties related to their organization, such as pore clustering, have not been thoroughly studied.

The process of bioceramic sintering can be prone to form a complex microarchitecture with high surface roughness and microporosity (Wilson et al., 2004; Champion, 2013). For example, the creation and distribution of micropores during sintering can create clusters of them with amounts in the order of 10^3^, which could be difficult to quantify with the traditional tools used in tissue engineering. Thus, a necessity appears to develop techniques to analyse these big data sets. These techniques can be used to relate these large micropore datasets and their influence on the mechanical and biological performance of bioceramics.

In this work, a novel method is used to asses pore clustering and to study the microarchitecture of CaP bioceramic particles as a model that presents both micro and macroporosity, driven by the research question: what are the geometrical properties of individual micropores in a CaP bioceramic, and how do they relate to each other? Material properties were studied by crystallographic phases and microstructural morphology. Hence, this study adds to the knowledge of CaP bioceramics and their use in regenerative medicine.

## Materials and Methods

### Bioceramic Material

In this work, the bioceramics used as a model were commercial Megagen Boneplus Eagle Eye Synthetic Bone Graft (http://www.imegagen.com).

**Figure 1:**
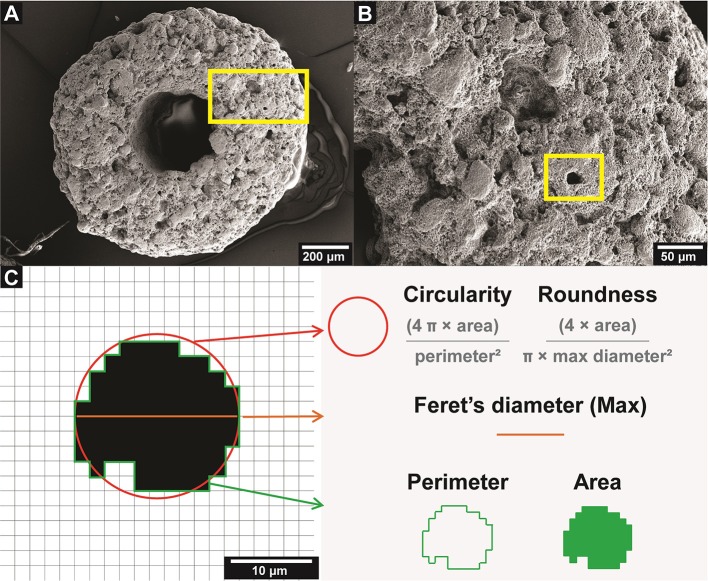
CaP bioceramic particle microarchitecture. **(A)** SEM micrograph of the bioceramic surface. **(B)** Area of interest marked in **(A)**, showing the microporosity of the bioceramic, the yellow square indicates a representative micropore. **(C)** Diagram of the five attributes measured for each micropore, based on Rueden et al. (2017).

These CaP bioceramics were used as received, which come in particles of a torus shape with a diameter of max 1 mm. According to the manufacturer, this material is made of a synthetic HA/β-TCP composite (60–40%), and each particle presents an interconnected micropore channel structure, while also having a central macro-pore (as shown in Figure 1).

### Microstructural Morphology and Phase Identification

The microstructural morphology of the bioceramic surface was analyzed by scanning electron microscopy (SEM). Samples were mounted on aluminum stubs with double-sided carbon tape and sputter coated with gold for 30 s at 45 mA (Denton Vacuum Desk V). Subsequently, SEM images were obtained (Jeol JSM IT300LV).

The crystallographic phase was determined by means of X-ray diffraction (XRD) (Bruker D8 Advance), with Cu Kα_1_ radiation (λ = 1.5406 Å) operated at 40 kV and 30 mA. The XRD patterns were recorded in the 2θ range of 10° to 80°, with a step size of 0.001° and step duration of 34 s. The recorded XRD spectra were identified by matching the spectra with ones based on the structural data of similar apatite bioceramics available in the Crystallography Open Database (COD). The phase composition analysis was done using the Rietveld Method (Bish and Howard, 1988).

### Assessment of Micropore Clusters of Cap Bioceramic Particles

A total of five (*n* = 5) bioceramic particles were analyzed by SEM imaging, obtaining more than 5 × 10^3^ micropores to be analyzed. The images were processed using ImageJ software (NIH, USA, version 1.51j8), with the aim of isolating only the micropores from the surface of the bioceramic particles, to perform the measurements and the subsequent analysis of clusters.

In order to isolate the micropores of each bioceramic particle, the threshold tool from ImageJ was used, in which the SEM microphotographs were converted to their corresponding binary images, where black pixels represent empty spaces (pores), following a similar protocol used previously (Xu and Chan, 2008). The validation of this porosity measurement was done using an accepted methodology (Xu et al., 2007).

For each of the more than 5 × 10^3^ isolated micropores (observations) the values of five geometrical attributes (variables) were determined: area, perimeter, circularity, roundness, and Feret's diameter (Walton, 1948). The area is expressed in μm^2^ and the perimeter in μm. The circularity and roundness are expressed in values between 0 (one line) and 1 (one circle and sphere, respectively). Feret's diameter represents the distance between two parallel lines that are tangential to the contour of the projection of a particle, in this case micropores, which allows having a measurement in μm of the diameter of the micropores that have an irregular shape (Merkus, 2009). Details of these parameters are shown in Figure 1.

Clustering Analysis was conducted by using the K-means clustering algorithm, which is a method that finds K vectors that represent a complete data set and aims to minimize the sum of the squared distances between all the points and the center of the cluster (Gonzalez and Tou, 1974; Pham et al., 2005). K-means was applied to a series of groups from 1 to 10, and inertia values were obtained (sum of the square distances of each cluster micropore to its centroid), which were used to determine the number of groups by means of the “Elbow” method. This method allows representing in a linear graph the inertia with respect to the number of clusters, indicating an inflection point and evaluating the maximal inflection. This point (elbow) indicates the optimal number of groups for the data set (Kaufman and Rousseeuw, 1990).

The validation of the number of clusters obtained was done by the Davies-Bouldin index (DBI), which is based on the fact that those algorithms that produce clusters with low intracluster distances (high intracluster similarity) and high intercluster distances (low intercluster similarity) will have a low DBI. Consequently, the clustering algorithm that generates a collection of clusters with the lowest value of this index was considered the best algorithm (Davies and Bouldin, 1979).

Finally, the analysis of clusters was deepened by relating the Feret's diameter of the micropores with their circularity (RStudio Inc., version 1.0.153), since it is known that the size and shape of the pores of a biomaterial is critical for cellular behavior (Akay et al., 2004; Zadpoor, 2015).

## Theory/Calculation

### Determination of Davies-Bouldin Index

The validation of the number of clusters obtained was done by means of the DBI (Davies and Bouldin, 1979), using the following equation:

$$\begin{array}{l} DB=\;\frac{1}{n}\overset{n}{\underset{i=1}{\sum }}\begin{array}{c} \max\\ i\neq j \end{array}\left(\frac{\sigma_{i}+\sigma_{j}}{d\left(c_{i},c_{j}\right)}\;\right) \end{array}$$

Where *n* is the number of clusters, *c*_*x*_ is the centroid of cluster *x*, σ_*x*_ is the mean distance of all observations in cluster *x* to the centroid *c*_*x*_, and *d(c*_*i*_,*c*_*j*_*)* is the distance between the centroids *c*_*i*_ and *c*_*j*_.

## Results

### Analysis of Cap Bioceramic Particle Microarchitecture

Since the architecture and microporosity of CaP biomaterials can influence its mechanical and biological performance (Rustom et al., 2019), an analysis of the bioceramic microarchitecture was carried out from SEM images. These data were processed using ImageJ, with the aim of isolating only the micropores from the surface of the bioceramic particles, to perform the measurements and the subsequent cluster analysis.

The micropores were individually analyzed, obtaining 5,338 observations from 5 bioceramic particles, in which the area, perimeter, circularity, roundness, and Feret's diameter were measured (Figure 1).

### Microporosity Cluster Determination of Cap Bioceramic Particles

Clustering is a data exploration technique that allows objects with similar characteristics to be grouped, in order to facilitate their subsequent processing. In this particular case, the K-means method was used.

**Figure 2:**
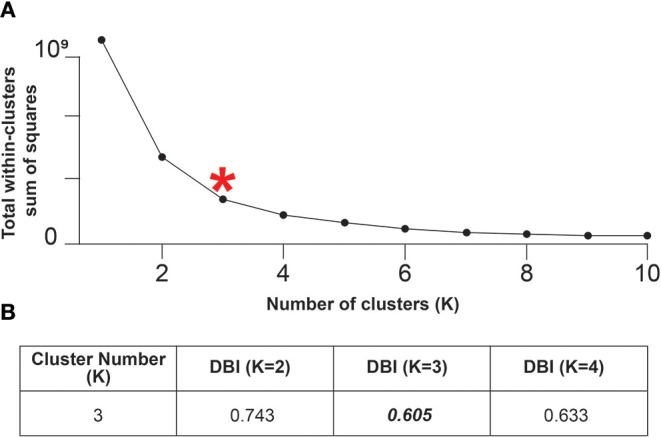
The CaP bioceramic particles show three micropore clusters. **(A)** Elbow method to determine the number of clusters in the 5,338 micropores data set, the “elbow” marked with the red asterisk suggests that the number of micropore clusters is three. **(B)** Davies-Bouldin Index (DBI) calculated considering the five attributes of the 5,338 micropores isolated from the five bioceramic particles, for which the *K*-value obtained by the elbow method, together with its closest neighbors, was used as an approximation. The lowest value of the DBI indicates the number of suitable clusters, in this case three.

K-means was applied to a number of clusters from 1 to 10, and the inertia values were obtained. Then, after using the Elbow method, the suggested number of clusters in these particles was three (*K* = 3). In order to confirm this result, the data were validated by means of the DBI, which showed that when comparing a *K* of 2, 3, and 4 (using the closest neighbors), a *K* = 3 gives the lowest DBI, and thus validates this result (Figure 2).

**Figure 3:**
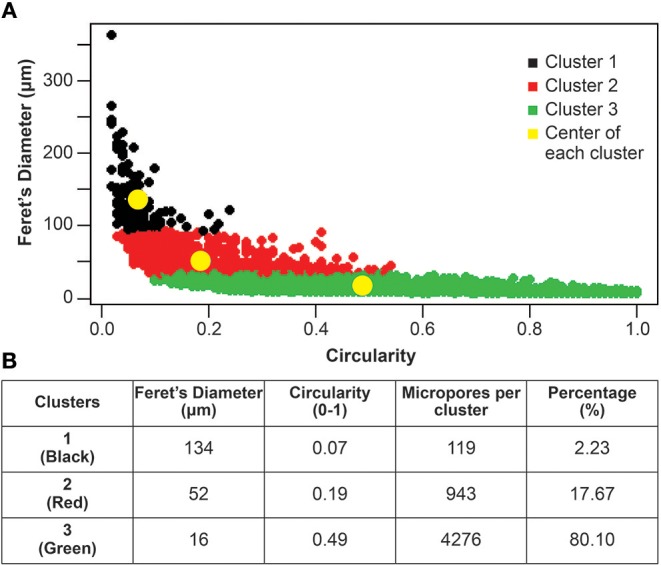
The three micropores clusters based on Feret's diameter and circularity. **(A)** The three clusters of micropores are highlighted in black (1), red (2), and green (3). The yellow dots indicate the centers of each cluster. **(B)** Table showing the Feret's diameter and the circularity of the centers of each cluster (yellow dots in **A**). It also indicates the number of micropores that belong to each cluster and their representative percentage within the bioceramic particles.

The analysis of clusters was deepened by relating the Feret's diameter of the micropores with their circularity. This analysis indicates that within these three clusters of micropores, the first cluster represents <3% of the micropores, has a Feret's diameter of more than 100 μm but a circularity generally lower than 0.1, being the least circular pores. The second cluster represents <20% of the micropores, has a Feret's diameter of about 50 μm and circularity near 0.2. While the third cluster represents most of the micropores (more than 80%), has a Feret's diameter of <50 μm and a wide range of circularity up to 1, being the most circular pores (Figure 3).

### Phase Identification of Cap Bioceramic Particles

In order to corroborate this CaP bioceramic crystallographic phases, their XRD pattern was indexed using standard cards by the COD. This bioceramic showed two phases, which matched with the card number PDF 86-0740, correlated to hydroxyapatite, and PDF 03-0713, correlated to Whitlockite, which corresponds to β-tricalcium phosphate (Jarcho et al., 1979).

**Figure 4:**
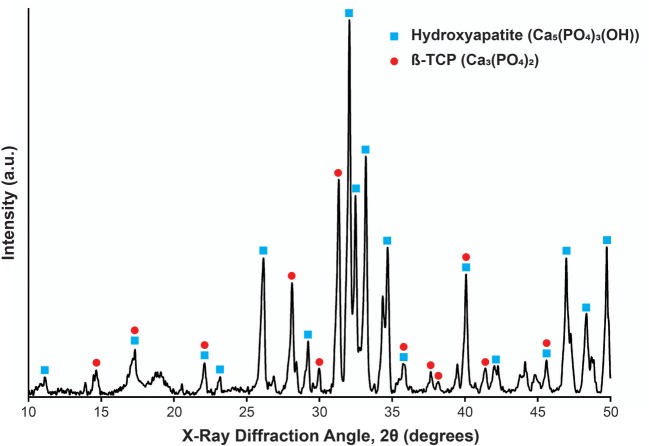
X-Ray diffraction pattern for the bioceramic particle used. The peaks corresponding to Hydroxyapatite and β-TCP are shown by blue squares and red circles, respectively. Peaks identification based on Lee et al. (2013).

The Rietveld composition analysis showed that this bioceramic is composed of 53.74% hydroxyapatite (Ca_5_(PO_4_)_3_OH) and 39.02% β-tricalcium phosphate (Ca_3_(PO_4_)_2_), with the rest of the components being other calcium phosphates or calcium oxide: 3.57% of CaP_4_O_11_ and 3.68% of CaO. Therefore, the main crystallographic phases of these bioceramic particles are indeed hydroxyapatite and β-tricalcium phosphate (Figure 4).

## Discussion

This research analysis shows that in these CaP bioceramic particles, three groups of micropores are distinguished by their area, perimeter, circularity, Feret's diameter and roundness. To the best of the author's knowledge, this is the first report on the analysis of microporosity clusters of CaP bioceramics particles with the described method, driven by the research question: what are the geometrical properties of individual micropores in a CaP bioceramic, and how do they relate to each other?

Given that on the one hand, the fabrication process of bioceramics leads to the generation of a complex microarchitecture (Wilson et al., 2004), which have an effect on their biological (Polak et al., 2013; Rustom et al., 2016) and mechanical properties (Pecqueux et al., 2010). And on the other hand, the generation of these large amounts of micropores requires techniques to analyse these big data sets. This study used CaP bioceramic particles as a model, to validate a novel method to analyse such microarchitecture in terms of micropore clustering, which has not been previously studied on this bioceramic (Conserva et al., 2011).

The microstructural and morphological properties of CaP bioceramics are related to biological and cellular performance, for example, it has been shown that the size and shape of HA microparticles have an influence on the osteogenic differentiation of MC3T3-E1 preosteoblasts (Xu et al., 2018). Furthermore, microporosity (pores of < 50 μm) plays a role in bone ingrowth and in the improvement of a scaffold mechanical properties (Rustom et al., 2019).

In this study, almost 20% of the micropores (clusters 1 and 2) have a Feret's diameter of 50 μm or more. In a study that used biphasic CaP bioceramics *in vivo*, these large pores have been shown to promote natural bone deposition and resorption processes (Lan Levengood et al., 2010b). The remaining 80% (cluster 3), corresponds to micropores of smaller size (20 μm or less). These small pores can have a higher capability to adsorb proteins *in vitro*, which can influence the adhesion and proliferation of human bone cells (Rouahi et al., 2006). Additionally, it has been shown that an increase in surface roughness significantly improves cell attachment and proliferation (Li et al., 2005), as well as improving the homogeneity of bone distribution by micropore-induced capillarity (Rustom et al., 2016).

The abovementioned pore properties, added to the existence of a central macropore in each of these particles, which could allow vasculogenesis, as shown for the macropores of other CaP bioceramics (Lan Levengood et al., 2011), make these particles suitable for bone tissue regeneration, with proper oxygen and blood supply. Besides, it is known that biphasic CaP bioceramics create a unique microenvironment that favors bone regeneration (Lobo and Arinzeh, 2010).

By using the presented methodology, future studies will be able to relate micropore geometry and clustering with biological performance of different CaP bioceramics in terms of adhesion, proliferation, or differentiation, among others. Consequently, an optimal set of microarchitectural parameters can be found, which will lead to a desired response in clinical therapies. Furthermore, this methodology could be applied to structures made of other materials, and fabricated by processes that could allow for major control of their microarchitecture for bone tissue engineering applications (Turnbull et al., 2017).

Within the limitations of this study, it is the surface microarchitecture of the CaP bioceramic and not the internal microarchitecture of the particle that was examined, which in future studies should be investigated to determine if there are variations in the characteristics of the micropores and, consequently, of the clusters. Likewise, it is necessary to analyze a larger number of particles, different manufacturing lots, and incorporate a more comprehensive and multi-scale analysis in order to integrate biology with the materials science paradigm (Roeder, 2010).

## Conclusions

In this study, a novel method based on data mining techniques was used to analyze micropore clustering in CaP bioceramic particles, and five geometrical parameters were assessed in each micropore (area, perimeter, circularity, Feret's diameter, and roundness). As a result, three clusters of micropores are described, in which almost 20% of the micropores (clusters 1 and 2) have a Feret's diameter of 50 μm or more, with the remaining 80% (cluster 3), having micropores of smaller diameter (20 μm or less).

This new methodology can be applied to advance the knowledge about CaP bioceramics by analyzing global micropore properties, such as pore clustering, and their relation with other properties of interest, such as mechanical or biological performance, for bone tissue engineering.

## Data Availability Statement

The datasets generated for this study are available on request to the corresponding author.

## Ethics Statement

The studies involving human participants were reviewed and approved by Universidad Adolfo Ibáñez. The patients/participants provided their written informed consent to participate in this study.

## Author Contributions

CM and JV planned the study. IB, VI, and JV performed the experiments. All authors contributed to the results analysis and RV, IB, CM, and JV contributed to the manuscript writing.

### Conflict of Interest

The authors declare that the research was conducted in the absence of any commercial or financial relationships that could be construed as a potential conflict of interest.
